# Genome Characterization of *Gordonia* Phage Amore2

**DOI:** 10.1128/mra.00537-22

**Published:** 2022-09-28

**Authors:** Caroline M. Lantis, Emily C. Mattison, Sarah E. Miller, Alexandria J. Williams, Alison E. Kanak

**Affiliations:** a Department of Biology, University of North Georgia, Dahlonega, GA, USA; Portland State University

## Abstract

Novel *Gordonia* phage Amore2 was isolated from Pittsburgh, Pennsylvania and infects Gordonia terrae 3612. Amore2 was placed into Actinobacteria cluster CS1. Its genome is 73,842 bp with 105 predicted open reading frames and 56.6% GC content. The closest similarity of Amore2 is *Gordonia* phage Austin, with a 98% nucleotide identity.

## ANNOUNCEMENT

Bacteriophages are viruses that infect bacteria (1). These viruses are evaluated in diverse fields of study from clinical implications to understanding micro levels of evolution. This work describes the *Gordonia* phage Amore2, which was isolated using the Science Education Alliance-Phage Hunters Advancing Genomics and Evolutionary Science (SEA-PHAGES) protocol (2).

Amore2 was isolated from soil located in Pittsburgh, Pennsylvania (40.441502 N, 80.002022 W). Gordonia terrae 3612 was used as a host for all of Amore2’s isolation and characterization procedures (3). PYCa liquid medium (4) was added to soil and incubated at 30°C for 24 h. The sample was filtered with a 0.22-μm syringe filter. The presence of phage was confirmed and phage were purified through multiple rounds of single-plaque isolation, and then amplified to a high titer to extract phage genomic DNA.

Phage genomic DNA was extracted from Amore2 lysates with a Wizard DNA extraction kit (Promega), according to the manufacturer’s instructions. A NEBNext Ultra II FS kit with dual-indexed barcoding was used to assemble a sequencing library. The Amore2 library was run on an Illumina MiSeq system. There were ~512,051 single-end 150-bp reads from the Amore2 library that, when assembled, provided ~988-fold coverage. These raw reads were assembled using Newbler v2.9 with default settings (5). The resulting single phage contig was checked for completeness, accuracy, and phage genomic termini using Consed v29 (6). The assembly had consistent coverage throughout and reads at the ends were continuous with reads at the other end, indicating a complete genome. Any consensus positions with >18% discrepant reads were manually inspected and corrected if necessary. Buildups of read starts were identified and used to determine packaging strategy and base 1 of the genome.

The genome was annotated using GeneMark v3.25 (7), NCBI BLAST v2.9.0 (8), Glimmer v3.02 (9), HHpred v3.2.0 (10), ARAGORN v1.2.38 (11), and Phamerator (https://phamerator.org). Default parameters were used unless otherwise specified. Hits with an E value of 10e^−10^ or less were considered acceptable. The Amore2 genome has ends with direct terminal repeats which are 179 bp long. Phamerator and GeneMark indicate that Amore2 has 105 open reading frames (ORFs), 26 of which have predicted functions. Only genes 10 through 41 are predicted to be transcribed in the forward direction, while all other genes are predicted to be transcribed in reverse. Phage with at least 50% nucleotide similarity are grouped into clusters and Amore2 was classified as a CS1 phage. All four CS1 phages have an average GC content of 56.7%. Interestingly, ORF 91 broke Amore2’s stream of synteny with bacteriophage Austin. Results of I-TASSER (12) protein analysis indicate ORF 91 codes for a protein with structure that is most similar to putative transcription regulators of the IclR family (13). The structural genes of Amore2 are contained in the left arm of the genome, while the right arm contains mostly genes with an unknown function. Amore2 is most genetically similar to Austin (GenBank accession no. MW534374), with 98% nucleotide identity determined via BLASTn alignment (8).

### Data availability.

Information on Amore2’s genome can be found in GenBank under the accession no. ON526973. Sequencing reads are part of the Sequence Read Archive with accession no. SRX15121734 under BioProject accession no. PRJNA488469.
